# Effects of Variety and Grape Berry Condition of *Vitis vinifera* on Preference Behavior and Performance of *Drosophila suzukii*

**DOI:** 10.3390/insects10120432

**Published:** 2019-11-30

**Authors:** Lisa Weißinger, Niklas Samuel, Michael Breuer, Caroline Müller

**Affiliations:** 1 Department of Chemical Ecology, Bielefeld University, Universitätsstr. 25, 33615 Bielefeld, Germany; lisa.weissinger@googlemail.com; 2 State Institute of Viticulture and Enology (WBI), Merzhauser Str. 119, 79100 Freiburg im Breisgau, Germany; kontakt@biologe-samuel.de (N.S.); Michael.Breuer@wbi.bwl.de (M.B.); 3 Wissenschaftliche Projektbearbeitung & Artenschutzrechtliche Begutachtung, Winzerstraße 3, 79292 Pfaffenweiler, Germany

**Keywords:** spotted wing drosophila, invasive species, host plants, insect behavior, *Vitis* variety, preference-performance, fruit damage

## Abstract

*Drosophila suzukii* is an invasive fruit pest and represents a potential economic threat to viticulture. After first observations of *D. suzukii* in Europe in 2008, research mainly focused on the evaluation of the host range and infestation risk for fruit and berry crops. However, the risk assessment of *D. suzukii* in viticulture has only recently started. Understanding the factors influencing preferences of *D. suzukii* for host species and varieties as well as offspring performance is essential to improve management strategies. We investigated the field infestation of different grape varieties across Baden-Wuerttemberg, southwestern Germany, between 2015 and 2018. Moreover, we performed dual-choice assays in the laboratory to investigate whether adults show preferences for certain varieties and whether offspring performance differs between varieties. Furthermore, we studied the impact of grape damage on choice behavior. Field monitoring revealed that *D. suzukii* show preferences for red varieties, whereas almost no oviposition occurred in white varieties. The results of dual-choice assays confirmed that *D. suzukii* preference and performance are influenced by grape variety and that flies preferred damaged over intact “Pinot Noir”, “Pinot Blanc”, and “Müller-Thurgau” berries. Overall, these findings may have important implications for winegrowers regarding cultivated varieties, grape health, and insecticide reduction.

## 1. Introduction

About ten years ago the Asian fruit fly *Drosophila suzukii* Matsumura (Diptera: Drosophilidae; spotted wing drosophila) was accidentally introduced into Europe and North America and nowadays it is a well-established economic pest across both continents [1,2,3]. A broad range of cultivated and wild soft-skinned fruit species have been identified as potential suitable hosts for this polyphagous generalist so far [4,5,6,7,8,9]. Recent studies indicate that *D. suzukii* shows preferences for certain fruit species and varieties but not all preferred hosts are suitable for larval development and are, thus, so-called dead-end hosts [4,5,10]. While previous research has mainly focused on the susceptibility of berry crops [11,12,13], little information is available on the infestation of grape berries, an only occasionally infested host [14,15,16], as well as on factors driving differences in susceptibility between host species and varieties [17,18,19]. The prevailing uncertainty about the risk of damage by *D. suzukii* arising for wine production has resulted in management strategies mainly based on the prophylactic use of broad-spectrum insecticides. However, these pesticides are known to seriously impact non-target organisms across trophic levels and can cause insecticide resistance, as found in several *Drosophila* species [1,6,20]. Therefore, more knowledge on the susceptibility of different grape varieties and factors influencing *D. suzukii* behavior is needed to contribute to the development of efficient and sustainable management strategies.

Unlike most other *Drosophila* species that attack overripe and decaying fruits, *D. suzukii* prefers oviposition into intact, still ripening fruits [2,4,21]. The unusual ability to puncture the skin of healthy immature fruit and to insert eggs into the pulp is based on one key adaption of *D. suzukii* females, the large, saw-like ovipositor [8,22,23]. In addition, the species has a short generation time, high fecundity, and a high dispersal potential [8,22,24]. With these characteristics, *D. suzukii* is a potential economic threat causing yield losses of up to 80%, especially to the small-fruit industry [2,13].

Various visual, physical, and chemical factors may affect *D. suzukii* preference behavior for different plant species and varieties and offspring performance in different fruits, such as volatiles, size, color, skin firmness and penetration force, maturity, sugar content, acidity, and nutrient content [16,17,25,26,27,28,29,30]. For example, *D. suzukii* utilizes visual cues such as fruit color as remote sensory information for host location [31]. Previous research suggests that fruits of different colors, including black, blue, pink, purple, orange, brown, and white are accepted for *D. suzukii* oviposition, with color preferences for red or purple over green [4,10]. According to the preference-performance hypothesis, female insects should have an oviposition preference for hosts on which offspring show the best performance [32,33]. For *D. suzukii*, positive relationships between adult preferences and offspring performance were indeed observed, e.g., in bioassays with wild and cultivated blueberries and in choice and no-choice assays using artificial diets with different protein-to-carbohydrate ratios [26,30]. In contrast, preference and performance did not match on different *Prunus* species [12] or when testing purees of different fruits [18].

Moreover, there have been few studies reporting that previously injured berries are preferred by *D. suzukii* for oviposition compared to intact berries, which does not necessarily lead to a better performance of the offspring in those berries compared to that in intact fruit [34,35,36,37]. For example, in cold-hardy grapes and muscadine grapes, *D. suzukii* laid more eggs and had a better offspring performance in damaged compared to intact fruits [3,37]. Holle et al. (2017) [36] found a higher infestation in injured blueberries, cherry tomatoes, and table grapes than in intact fruit, whereas adult emergence was only higher in injured grapes and tomatoes.

The present study focused on the risk assessment of different grape varieties with regards to infestation by *D. suzukii*. Therefore, we investigated the susceptibility of grape varieties in several viticultural districts across Baden-Wuerttemberg, southwestern Germany, during the seasons from 2015 to 2018. Based on these data, we picked five cultivars of different color and susceptibility for more in-depth analysis in the laboratory. With those cultivars, we tested whether *D. suzukii* adults show similar behavioral preferences for certain varieties in dual-choice assays in the laboratory as those found in the field and how the offspring develop on these cultivars. Furthermore, we investigated whether grape damage may turn less accepted varieties more susceptible and whether such damage has an effect on both preferences of adult *D. suzukii* and the performance of their offspring.

## 2. Materials and Methods

### 2.1. Monitoring of Field Infestation

To assess the susceptibility of different grape cultivars a weekly grape berry monitoring was conducted in various viticultural districts (Markgräflerland, Kaiserstuhl, Tuniberg, Freiburg, Ortenau, and Kraichgau) across Baden-Wuerttemberg, southwestern Germany, during the seasons from 2015 to 2018. From the beginning of veraison until harvest grape clusters of different red (in total 27) and white (in total 12) varieties were collected randomly from different vineyards and directly transported to the laboratory. Furthermore, winegrowers all across the region submitted berry samples to our institute (State Institute of Viticulture and Enology, Germany) for scoring of the infestation status by *D. suzukii*. Due to the fact that white varieties such as “Müller-Thurgau” and “Pinot Blanc” showed hardly any or no infestation in 2014 and 2015, these varieties were not further recorded in the following seasons. For these reasons, sampled varieties and the number of sampled sites varied among years (for details see Table S1). From each of the collected and submitted grape clusters, fifty randomly selected, sound berries were scanned under the stereomicroscope for *D. suzukii* eggs to determine the infestation status within 24 h after sampling. The eggs are detectable on the fruit surface by the two respiration filaments (extensions of the chorion) [10,23]. For field infestation data, we calculated the weekly mean (±SD) proportion of *D. suzukii* eggs detected per berry sample (= infestation severity in %) per variety and year (Table S1).

### 2.2. Insect Rearing Conditions and Colony Maintenance

Individuals of *D. suzukii* used in dual-choice assays originated from a laboratory population kept at State Institute of Viticulture and Enology, which was established in 2014 from different infested wild berries collected in the viticultural district Kaiserstuhl (48°4′51″ N, 7°40′14″ E; 556.6 m a.s.l.; Baden-Wuerttemberg, southwestern Germany). The colony was maintained for over 50 generations and was genetically refreshed with flies from a laboratory rearing of Julius Kühn-Institute in Dossenheim (Germany) in 2016 by crossing insects from both colonies. This fly colony was maintained with a sugar-water source (5% sucrose) and a mix of brewer’s yeast (Masterdog, Schwieberdingen, Germany) and dry sugar (1:1) in rearing cages (30 × 30 × 30 cm, Bugdorm-1, Megaview, Taiwan). The cages were kept in a climate chamber (Viessmann, Allendorf, Germany) at 24 °C, 60–70% r.h. and a photoperiod of L16:D8. As a food source and an oviposition medium, flies were provided with “San Michele medium” [1.3 L water, 30 g sugar, 142 g cornmeal (Alnatura, Freiburg, Germany), 20 g soy flour (Alnatura), 34 g brewer’s yeast, 11.2 g agar (Agar Kobe I, AppliChem GmbH, Darmstadt, Germany), 5 g vitamin mixture (Vanderzant, MP Biomedicals, USA), 9.4 mL propionic acid (Roth, Karlsruhe, Germany), and 3.34 g nipagin (Alfa Aesar, Thermo Fisher GmBh, Karlsruhe, Germany)] [34]. The medium was filled in Petri dishes (Ø 9 cm), which were placed in the cages for oviposition. After 2–3 days Petri dishes were removed, sealed with perforated lids and stored in a climate chamber under the same conditions as stated above. When adult flies emerged, Petri dishes were placed in new rearing cages and lids were removed. Adult female flies used in dual-choice assays were tested between 6 and 12 days after adult emergence because we found the highest responsiveness in females of this age in preliminary assays.

### 2.3. Grape Varieties and Grape Condition for Laboratory Tests

Three red grape varieties, “Acolon”, “Regent”, and “Pinot Noir”, and two white varieties, “Müller-Thurgau” and “Pinot Blanc”, were selected to assess *D. suzukii* preference behavior as well as their performance in laboratory dual-choice assays. These varieties were chosen because they are commonly grown in the viticultural districts of Baden-Wuerttemberg and show different ripening time points (early season varieties: “Acolon”, “Regent”, and “Müller-Thurgau”; late-season varieties: “Pinot Noir” and “Pinot Blanc”) as well as different susceptibility to *D. suzukii* based on results of the field sampling (susceptible varieties: “Acolon”, “Regent”; less susceptible variety: “Pinot Noir”; not susceptible varieties: “Müller-Thurgau” and “Pinot Blanc”; see results, Table 2; Figure 2).

Thus, we compared the fly behavior towards meaningful pairs of red vs. white and red vs. red varieties. Two early season, red and more susceptible varieties (“Acolon”, “Regent”) were tested against one early season, white and not susceptible variety (“Müller-Thurgau”). One late season, red and not susceptible variety (“Pinot Noir”) was tested against one late-season, white and not susceptible variety (“Pinot Blanc”). Furthermore, two red (“Regent”: early season and susceptible vs. “Pinot Noir”: late season and not susceptible) varieties were tested against each other (Table 1) at an overlapping time point of ripeness.

Grape berry clusters of each variety used for insect bioassays were randomly collected from vineyards that received the same cultivation practice and were not treated with insecticides. Grape berries were picked when ripe and stored for a maximum period of 24 h before starting dual-choice assays. Prior to the dual-choice assays, berries were examined under the dissecting microscope (Zeiss Discovery V20, Jena, Germany) to exclude pre-infestation and to discard visibly damaged and overripe berries. To investigate whether varieties that were less accepted in choice tests may turn more susceptible when being damaged, the effects of berry condition on preferences and performance of *D. suzukii* were tested using “Pinot Noir”, “Müller-Thurgau”, and “Pinot Blanc.” Half of the intact grape berries were artificially damaged at the surface with a scalpel. The wounds were approximately 2–3 mm wide.

### 2.4. Laboratory Dual-Choice Assays

To assess differences in susceptibility of certain grape varieties and berry conditions to *D. suzukii* under controlled conditions, behavioral preferences (location and oviposition) of *D. suzukii*, as well as offspring performance (emergence rate), were evaluated in dual-choice assays. Two sets of dual-choice assays were performed: (1) dual-choice tests between intact berries of different varieties and (2) choice tests between intact and damaged berries of the same varieties. In total, seven combinations were tested, and assays were replicated 10–40 times in 2016 and 2017 (for detailed information about the tested combinations and replicates see Table 1).

**Figure 1:**
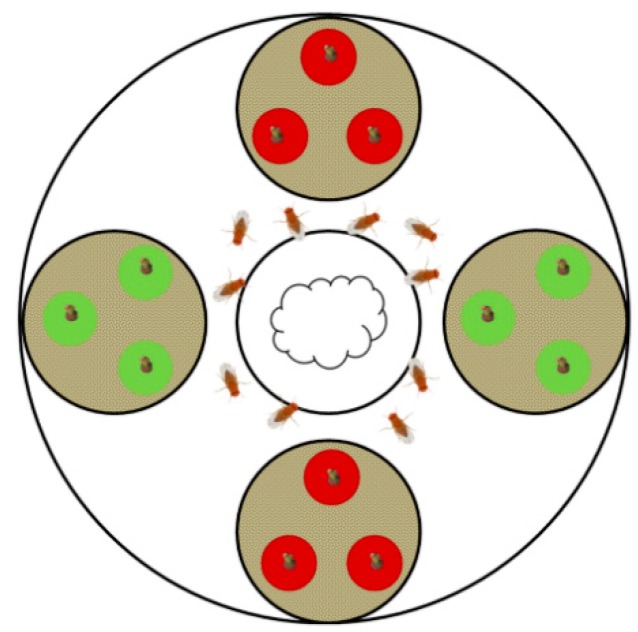
Top-view of the test arena used in dual-choice bioassays. Ten female flies were released in the center of each arena. Two times three berries per choice option (variety or condition) were placed in two small Petri dishes on opposite sides of the arena. The bases of the test berries were embedded in sand to prevent the flies from hiding and to keep the berries in place.

For the dual-choice assays, test arenas were designed in which ten female flies were given the choice between two options, respectively (Figure 1). Each of the test arenas (glass Petri dish, Ø 19 cm) contained five smaller Petri dishes (Ø 6 cm). One of these small dishes was placed in the center of the arena and contained a water-soaked cotton wick to provide a water source, the other four dishes contained each three test berries. To prevent the flies from hiding under the berries and to keep the berries in place, the bases of the berries were embedded in sand (Silex Spielsand, Globus Baumarkt, Herbolzheim, Germany) with stems facing upwards. Two times three berries per choice option (variety or condition) were placed in two small Petri dishes on opposite sides of the arena (Figure 1). Test arenas were closed with a gauze lid (1 mm mesh). Each dual-choice assay was replicated 10 times (= 10 arenas) simultaneously in a climate chamber (24 °C, 60–70% r.h., photoperiod L16:D8) per experimental date. Arena positions and orientations were randomized for each experimental date.

Before starting the dual-choice assays a small sample of berries was offered in rearing cages to test for the motivation of *D. suzukii* and acceptance of berries. As soon as infestation took place, dual-choice assays were started. Additionally, the sugar content (°Brix) of ten randomly selected berries of the same grape cluster used in the dual-choice assays was determined for each variety using a handheld, temperature-compensated refractometer (VWR, Darmstadt, Germany). Both infestation and sugar content were determined within 24 h after collection of the berries in the field.

Flies were removed from rearing colonies approximately 1–2 h before the start of each assay. To select females only, we used a minimal invasive method for anesthetizing the flies by placing them on ice. After females were released into the center of the arenas (10 females per arena), their position was recorded at three time points, after 5 h, 7 h, and 24 h (location preference). After 24 h, flies were removed and eggs deposited on the berries were counted (oviposition preference) under the dissecting microscope, summing up numbers of eggs that were placed on the berry surface as well as eggs that were already dead due to wound healing of the berry. Most females were still alive after the experimental period (mortality less than 6% over all assays). In addition, in 2017 all test berries were stored in plastic cups (500 mL), incubated at 24 °C, 60–70% r.h., photoperiod L16:D8, and scored every 5–7 days for adult emergence (emergence rate) as a measure for offspring survival for a period of about 30 days.

### 2.5. Statistical Analysis

All statistical analyses were performed in R version 3.3.2 [38]. To analyze the effects of grape variety (“Acolon”, “Regent”, “Müller-Thurgau”, “Pinot Noir”, and “Pinot Blanc”) and berry condition (intact vs. damaged) on location and oviposition preferences of *D. suzukii*, separate linear mixed-effects models (LMMs) with a Gaussian error distribution were performed for each test combination (R package: lme4; [39]).

For statistical analyses of the location preference data, the number of flies sitting on or within a radius of <1 cm around the berries of the same variety or berry condition (n = 6) after 24 h was summed up for each test arena. We calculated seven LMMs (one per test combination), which comprised the number of *D. suzukii* females per choice option as the response, the factor variety or berry condition as fixed effects, and the test arena, as well as date nested within year as random effects.

For the oviposition preference data, only the number of successfully deposited eggs (vertically inserted and visible egg filaments) in berries of the same choice option (variety or berry condition) was calculated per arena. We computed seven LMMs (one per test combination), which comprised the number of eggs per choice option as the response, the factor variety or berry condition as fixed effects, and the test arena, as well as date nested within year as random effects.

To choose the appropriate error distribution (“family”) for LMMs, it was assured that residuals exhibited variance homogeneity and normal distribution of residuals by means of visual inspection [40]. We applied step-wise backward model selection based on chi-square likelihood ratio tests to obtain *p*-values for the effects of all predictor variables [41].

For statistical analyses of the adult emergence data of 2017, we calculated the proportion of emerged adults from deposited eggs per choice option and arena. A non-parametric Mann–Whitney U test was performed to determine statistical differences between choice options.

## 3. Results

### 3.1. Field Infestation 2015–2018

**Figure 2:**
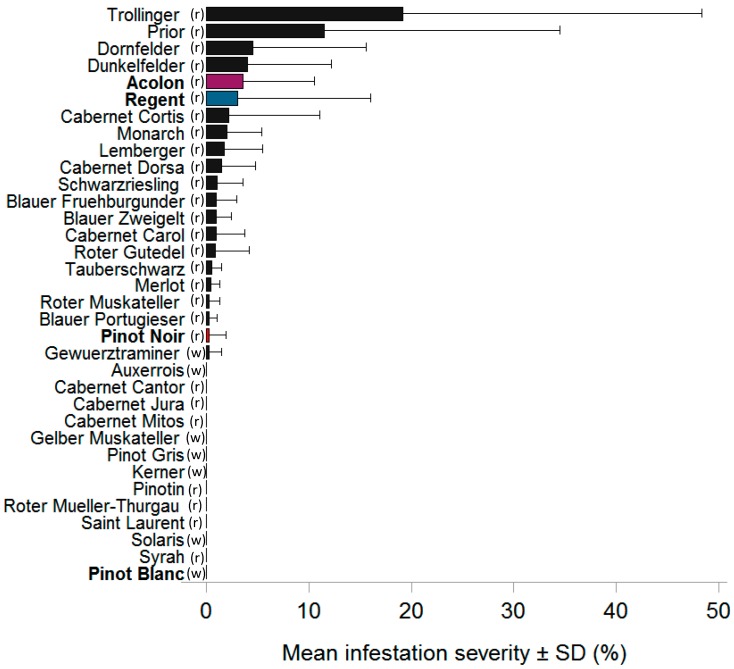
Weekly mean (±SD) *Drosophila suzukii* infestation severity (number of eggs per berry sample (50 berries) in %) of red (r) and white (w) grape varieties in vineyards of various viticultural districts across Baden-Wuerttemberg (Southwestern Germany), in 2016. Means are based on different sample numbers (lowest: n = 1, highest: n = 201; see Supporting information Table S1). Varieties used for laboratory bioassays are indicated in bold, except for “Mueller-Thurgau”, which was not sampled in 2016.

Grape berry monitoring of different grape varieties growing in the viticultural districts across Baden-Wuerttemberg revealed overall a mean infestation severity across all investigated grape varieties of less than 7% in each of the study years, being highest in 2017 and lowest in 2018 (Table S1). However, *D. suzukii* flies showed varietal preferences. All other red varieties were infested except “Cabernet Cantor”, “Cabernet Jura”, “Cabernet Mitos”, and “Saint Laurent”. In every year of observation, samples of the varieties “Trollinger”, “Dornfelder”, “Lemberger”, “Portugieser”, “Rotel Gutedel”, “Schwarzriesling”, “Regent”, and “Pinot Noir” displayed infestation with *D. suzukii* eggs (Table S1). In 2016, chosen as an example, infestation severity was highest in the red varieties “Trollinger” and “Prior”, followed by “Dornfelder”, “Dunkelfelder”, “Acolon”, and “Regent” (Figure 2). In contrast, in the white varieties, no oviposition was found except for samples of “Gewürztraminer” (2015 and 2016) and “Müller-Thurgau” (2015), which showed an infestation severity of less than 0.5% (Table S1). Overall, there was a high variation within, as well as across years (Figure 2; Table S1).

When comparing the five selected, commonly grown varieties, the two early-season varieties (“Acolon” and “Regent”) similarly displayed the highest level of oviposition, followed by “Pinot Noir” which displayed an infestation severity of less than 2% (Table 2). Samples of the white variety “Müller-Thurgau” only showed infestation in 2015 with less than 0.5%, whereas no eggs were laid in berries of “Pinot Blanc” at all (Table 2).

### 3.2. Influences of Grape Variety on Preference, Oviposition, and Emergence of Drosophila suzukii in the Laboratory

**Figure 3:**
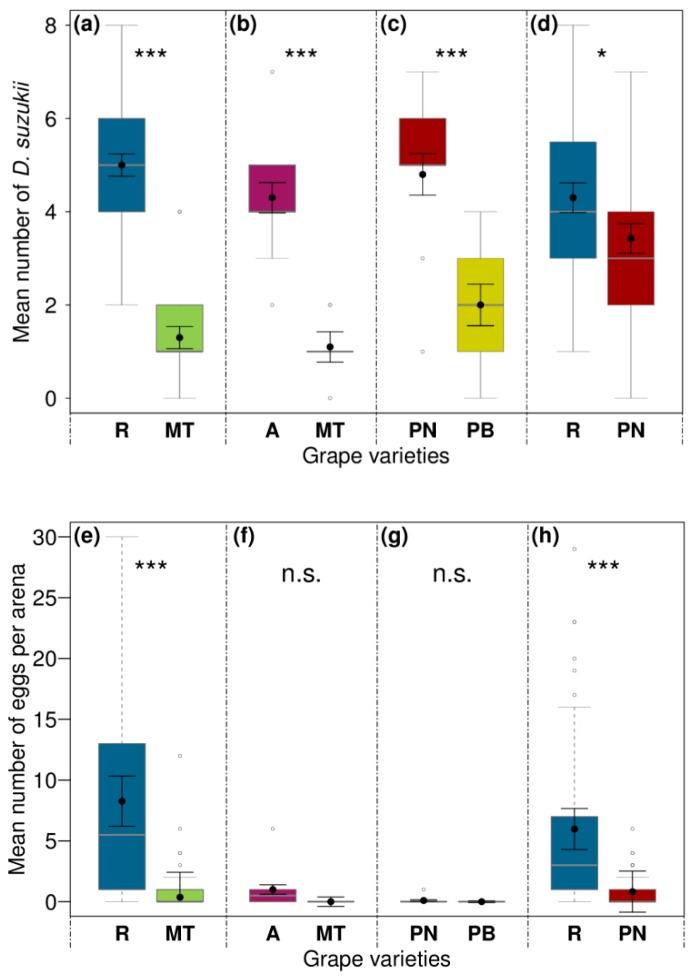
Effects of the grape variety on preferences of *Drosophila suzukii*. Mean fly number on grape berries or on respective Petri dishes (location preference; **a**–**d**) and the number of laid eggs per grape variety per arena (oviposition preference; **e**–**h**) in dual-choice bioassays after 24 h. Adult female flies were given the choice between berries of (**a**) “Regent” (R) vs. “Müller-Thurgau” (MT) (n = 30), (**b**) “Acolon” (A) vs. MT (n = 10), (**c**) “Pinot Noir” (PN) vs. “Pinot Blanc” (PB) (n = 10), and (**d**) R vs. PN (n = 40). Asterisks denote significant differences (n.s. not significant (*p* > 0.05), * *p* < 0.05, *** *p* < 0.001) among tested grape varieties (analyzed with LMMs; for statistical results see Table 3). Medians (grey lines) are presented with interquartile ranges (boxes), whiskers extend to the maximum and minimum values within the 1.5-fold interquartile range and outliers are shown as open circles. Black dots with error bars represent the least square means with their standard errors extracted from the LMMs, accounting for the specific error distribution of the response factor and for random effects.

One day after the set-up of the experiment, 69% of *D. suzukii* test females had made a choice for one of the two offered grape varieties. The number of flies sitting on berries (= location preference) was significantly affected by the grape variety in all four dual-choice assays after 24 h (Figure 3a–d and Table 3). In assays with red against white varieties, significantly more flies preferred “Regent” or “Acolon” over “Müller-Thurgau” (Figure 3a,b), and “Pinot Noir” over “Pinot Blanc” (Figure 3c). When given the choice between two red varieties, more flies were located on “Regent” than on “Pinot Noir” (Figure 3d).

The number of laid eggs was significantly affected by the grape variety in dual-choice assays between “Regent” vs. “Müller-Thurgau” or “Pinot Noir”, with flies showing oviposition preferences for “Regent” (Figure 3e,h and Table 3). Females laid about six times as many eggs on “Regent” compared to “Müller-Thurgau” and about three times as many eggs on “Regent” compared to “Pinot Noir.” A slight trend towards oviposition preferences for “Acolon” over “Müller-Thurgau” was found, although this was not significant (*p* < 0.1) (Figure 3f and Table 3). There were no statistical differences between the number of eggs laid on “Pinot Noir” and “Pinot Blanc”, with no oviposition at all on “Pinot Blanc” and almost none on “Pinot Noir” (Figure 3g and Table 3).

There were no differences in adult emergence rate from eggs deposited in berries of “Regent” vs. “Müller-Thurgau” tested in 2017, whereas from infested “Regent” berries about ten times more adults emerged than from infested “Pinot Noir” berries (Table 4).

### 3.3. Influences of Berry Condition on Preference, Oviposition, and Emergence of Drosophila suzukii in the Laboratory

**Figure 4:**
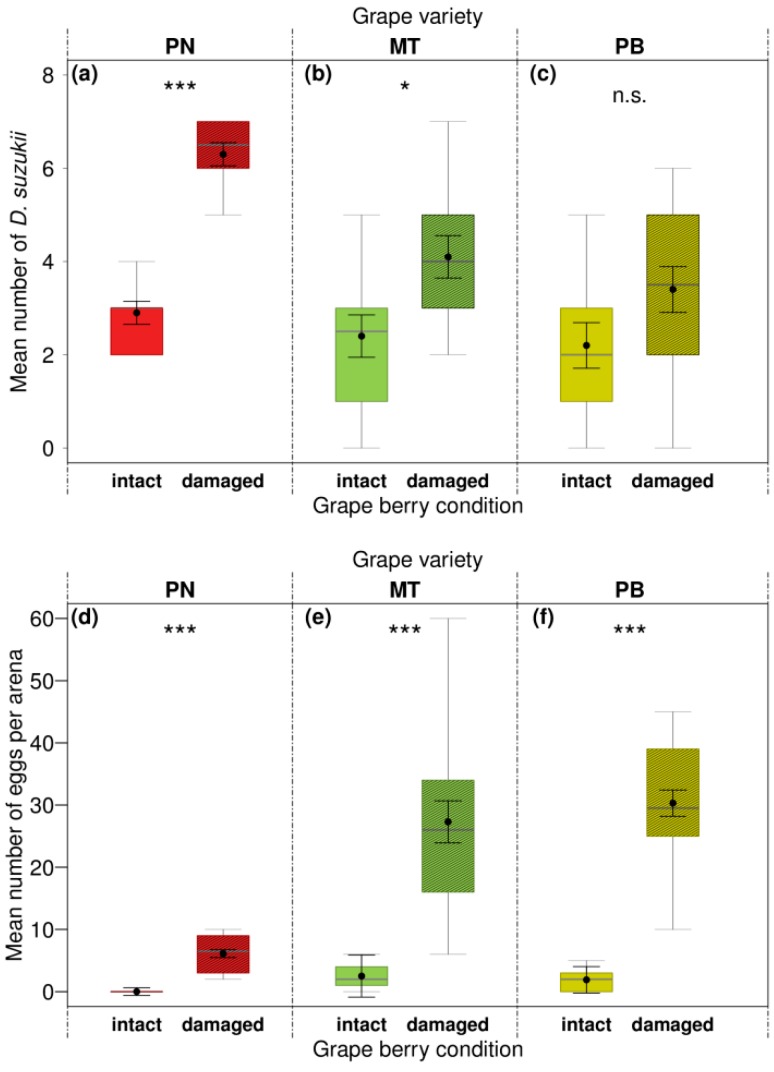
Effects of grape berry condition on preferences of *Drosophila suzukii*. Mean fly number on grape berries or on respective Petri dishes (location preference; **a**–**c**) and the number of laid eggs per grape berry condition (oviposition preference; **d**–**f**) in dual-choice bioassays after 24 h. Adult female flies (n = 10 per arena) were given the choice between intact and artificially damaged berries of “Pinot Noir” (PN), “Müller-Thurgau” (MT), and “Pinot Blanc” (PB; n = 10 replicates per test). Asterisks denote significant differences (n.s. not significant (*p* > 0.05), * *p* < 0.05, *** *p* < 0.001) among tested berry condition (analyzed with LMMs; for statistical results see Table 5). For details of boxes, error bars, and statistics see the legend of Figure 3.

One day after the set-up of the experiment, 71% of *D. suzukii* test females had made a choice for one of the two offered berry conditions. When given the choice between intact and damaged berries of “Pinot Noir” or “Müller-Thurgau”, flies significantly preferred to reside on damaged over intact berries (Figure 4a,b and Table 5). Likewise, in assays with “Pinot Blanc” flies tended to prefer damaged over intact berries, but this difference was not significant (*p* < 0.1) (Figure 4c and Table 5).

Female *D. suzukii* showed significant oviposition preferences for damaged berries in all three dual-choice assays (Figure 4d–f and Table 5). In intact “Pinot Noir” berries, no eggs at all were laid (Figure 4d). About 11 times as many eggs were observed on damaged compared to intact “Müller-Thurgau” and about 16 times as many eggs on damaged compared to intact “Pinot Blanc” berries. The emergence rate of adult flies was 15 times higher from eggs deposited in damaged than in intact “Müller-Thurgau” and about 7 times higher from eggs laid on damaged compared to intact “Pinot Blanc” berries (Table 4).

## 4. Discussion

Both field observations and laboratory experiments revealed that the infestation of investigated grape varieties by *D. suzukii* is almost exclusively limited to red varieties and occurs only in certain economically less relevant varieties. Location and oviposition preferences were found to be affected by the grape variety in dual-choice assays. Moreover, damage of berries from little accepted varieties turned these more susceptible. Importantly, we show that the pure presence of *D. suzukii* does not necessarily result in oviposition or successful offspring performance, which is in line with previous research on grapes [3,42] and in another fruit [12].

Based on our four-year field monitoring data, we compiled a ranking of grape infestation severity indicating that the three main economically important grape varieties “Pinot Noir”, “Müller-Thurgau” and “Pinot Gris” of the Baden wine region are only at very low risk of infestation by *D. suzukii* (Figure 2 and Table S1). Only some of the 39 investigated grape varieties grown in our study area, and thereof only red varieties, may be susceptible to *D. suzukii*. In contrast, white varieties were found to be hardly or not susceptible to *D. suzukii* oviposition (Table S1). Previous studies also identified red-colored fruits of different species or varieties to be preferred for *D. suzukii* oviposition [10,14,27]. However, it is known that *D. suzukii* is able to accept fruits of various colors for oviposition [4]. Our results are well in accordance with monitoring data from other regions, e.g., Rheinland-Pfalz, Germany [28], and Switzerland [43], in which partly the same grape varieties were investigated. However, in Switzerland Cahenzli and Daniel (2016) [44] reported medium susceptibility of some white varieties (e.g., “Solaris”, “Johanniter”, and “Bronner”) in no-choice tests. In Virginia, USA, Shrader et al. (2019) [15] found the highest *D. suzukii* oviposition in a white grape variety (“Viognier”) in choice tests. These white varieties may have other traits that turn them highly susceptible or alternatively, fly populations in these regions may respond differently. Overall, the field infestation severity of grapes in the present study appeared to vary strongly within and across years, which might be explained by seasonally changing environmental conditions, such as temperature, humidity, composition of the surrounding landscape and microclimatic conditions of sampling sites, known to potentially influence *D. suzukii* abundance [42,45,46,47].

Dual-choice assays confirmed that *D. suzukii* females show similar behavioral preferences for certain varieties in the laboratory as those found in the field. We observed significant location preferences of *D. suzukii* for red over white varieties. Our approach also revealed a slightly higher preference of *D. suzukii* to rest on “Regent” compared to “Pinot Noir” berries, although both varieties are red-colored (Figure 3a–d and Table 3). Interestingly, analysis of the oviposition preferences revealed that the physical presence of the fly alone is not enough to draw conclusions about the grape infestation. Only “Regent” berries were clearly preferred for oviposition compared to other varieties. In contrast, “Pinot Noir” was not accepted for oviposition by *D. suzukii*, although flies preferred to reside on this variety compared to “Pinot Blanc” (Figure 3e–h and Table 3). Likewise, “Acolon” attracted more females than “Müller-Thurgau”, but final oviposition was rather low in the first, and almost zero in the latter variety. Thus, while for the first attraction color, most likely together with volatiles [25], may be important cues for attraction, additional physical, and chemical cues determine the final host acceptance for oviposition [30], which should be investigated in more detail in future studies. In this regard, fruit firmness or skin penetration force are discussed to play a relevant role in *D. suzukii* oviposition ability, finding decreasing infestation with increasing penetration force [15,21,45]. In contrast, there are studies that confirmed higher oviposition rates in firmer berries [26] or no clear correlation between the two parameters [7,35]. In line with these findings, we likewise did not find a relation between force needed to penetrate berry surfaces, measured only for varieties tested in 2017 (data not shown), and oviposition preference in the studied grape varieties. Additionally, the physiological state of the females, as well as intraspecific competition, should be considered as factors potentially influencing oviposition preferences [25,26].

In line with other studies, offspring performance, here measured as adult emergence rates, tended to be rather low for all grape varieties [14,37,48]. Offspring performance was only assessed in dual-choice assays between varieties including “Regent” and appeared to be similar in choice assays with “Regent” against “Müller-Thurgau”, whereas the adult emergence rate was much higher in berries of “Regent” compared to “Pinot Noir”. The lower offspring performance in berries of “Regent” in the test against “Müller-Thurgau” may be explained by the fact that the number of eggs per berry tended to be higher than in the latter test, in which berries of “Regent” were tested against “Pinot Noir.” Thus, the adult emergence rate may decrease with increasing egg density due to intraspecific competition [18]. Variation in offspring performance among these two dual-choice assays may also be explained by potential differences in the microbial community on the surface of single grape berries [18,49]. Finally, subtle differences in fruit chemical composition and potentially also physical properties [26] influence the overall development of larvae in the fruits. For example, sugar content has been previously described to be a relevant parameter for the development of *D. suzukii* [10]. Previous studies revealed that there is no correlation between oviposition or performance with measured sugar contents (°Brix) [3,26]. In our study, only ripe berries of tested varieties were used for bioassays. °Brix levels were quite similar among all berries (Table 1), hence it might not have been a parameter that influenced differences in susceptibility, but the composition of sugars and of amino acids most likely differs between the varieties. The higher survival (i.e., adult emergence rate) in damaged versus intact fruits may indicate that berries with skin wounds offer a more suitable environment to eggs, larvae, and pupae, potentially due to higher oxygen availability in damaged fruits, but this needs further exploration.

To our knowledge, the combination of location, oviposition preferences, and offspring performance has rarely been studied in *D. suzukii* up to now. Our data indicate that the preference-performance hypothesis cannot be supported for all tested grape varieties. Location and oviposition preference were positively correlated for “Regent” compared to “Müller-Thurgau” but the relative adult emergence rates of the offspring did not match the initial preferences observed. Females of *D. suzukii* preferred to reside on “Pinot Noir” over “Pinot Blanc”, while in both almost no oviposition took place. A positive relationship of all three tested parameters was only found in the choice assay between “Regent” vs. “Pinot Noir**”**. Our findings are in line with Alhmedi et al. (2019) [12] who observed that flight activity, egg-laying behavior, and offspring performance of *D. suzukii* do not match on two *Prunus* species. Olazcuaga et al. (2019) [18] found no correlation between the preference and adult emergence in choice tests with the artificial medium of 12 different fruit species. Various polyphagous insect species, especially species becoming invasive, have been found not to conform to the preference-performance hypothesis [4,50]. This might be partially explained by other factors such as host plant abundance, predation avoidance, or natal host plant experience influencing females’ host selection [12,51].

The current work further demonstrates the crucial role pre-damage of grape berries is playing for *D. suzukii* infestation, by turning initially less accepted fruits more susceptible. Location and oviposition preference behavior were affected by berry condition particularly in the red grape variety with more flies resting and higher egg numbers oviposited on damaged compared to intact berries (Figure 4). Damaged berries may release more volatiles and thus be more attractive to the flies than intact berries [52]. Furthermore, injury allows direct and easier access of *D. suzukii* to fruit pulp. Consequently, varieties being unsusceptible to *D. suzukii* may become suitable hosts when injured. Adult emergence was also found to be strongly affected by pre-damage of the berries with successful offspring development and higher offspring numbers in damaged compared to intact berries of both white varieties. Our results are in line with previous research on the effects of berry condition on preferences and performance of *D. suzukii* in grapes [3,14,15,37] as well as in other fruits [34,36,52]. All these results raise the question of whether *D. suzukii* flies are causing primary damage in grapes or whether they are, at least sometimes, just a secondary pest taking advantage of previous damage, e.g., due to preceding feeding by wasps, damage by birds, disease, hail, or cracking caused by rain events [3,16].

## 5. Conclusions

Understanding the factors driving host use of *D. suzukii* is of great importance to provide growers with effective and sustainable management strategies, in which usage of pesticides should be reduced to avoid further loss of (beneficial) insect species. Our results show that *D. suzukii* oviposition on intact berries of certain grape varieties is possible but overall very low infestation and adult emergence rates suggest that intact grape berries are not very suitable hosts for *D. suzukii*. Moreover, the physical presence of *D. suzukii* on grape varieties such as Pinot Noir does not necessarily result in grape damage, which is of particular interest for winegrowers who observe *D. suzukii* in their vineyards. Preventive strategies for reducing pest pressure and pre-damage of grapes, such as the selection of less susceptible varieties, sanitation, and agronomic practices improving plant health, are alternative methods to currently recommended insecticides applications.

## Figures and Tables

**Table 1:** Combinations of grape varieties or berry conditions exposed to *Drosophila suzukii* females for 24 h in dual-choice arena bioassays. Relative density (in °Brix) of ten pooled and crushed berries of the same grape bunches as used in choice bioassays.

Test	Grape Varieties (V)	Combination Tested		Replicates (Arenas)	Year	Brix	
		V1	V2			V1	V2
1	Red vs. white	Regent	Müller-Thurgau	10	2016	20.5°	19.8°
				10	2017	20.5°	18.4°
				10	2017	19.8°	17.7°
2		Acolon	Müller-Thurgau	10	2016	18.8°	19.8°
3		Pinot Noir	Pinot Blanc	10	2016	23.8°	22.4°
4	Red vs. red	Regent	Pinot Noir	10	2016	21.4°	24°
				10	2016	20.9°	22.8°
				10	2017	22.4°	20.2°
				10	2017	21.2°	23.5°
	Berry condition						
5	Pinot Noir	intact	injured	10	2016	24°	
6	Müller-Thurgau	intact	injured	10	2017	17.7°	
7	Pinot Blanc	intact	injured	10	2017	21.2°	

**Table 2:** Mean (±SD) *Drosophila suzukii* infestation severity (number of eggs per berry sample (50 berries) in %) and calendar week (cw) of first oviposition on five selected grape varieties “Acolon”, “Regent”, “Müller-Thurgau”, “Pinot Noir”, and “Pinot Blanc” in the field. “N/A” = data not available; “ - ”= no calendar week of first oviposition due to zero infestation (0).

Year		Grape Varieties				
		Acolon	Regent	Pinot Noir	Müller-Thurgau	Pinot Blanc
2015	Severity (%)	0	1.58 ± 5.47	0.44 ± 1.68	0.30 ± 0.96	0
	First oviposition (cw)	-	36	36	38	-
2016	Severity (%)	3.57 ± 6.96	3.01 ± 13.03	0.29 ± 1.61	N/A	0
	First oviposition (cw)	33	34	36	N/A	-
2017	Severity (%)	2.60 ± 7.23	2.13 ± 6.82	1.23 ± 6.87	0	N/A
	First oviposition (cw)	33	33	36	-	N/A
2018	Severity (%)	0.04 ± 0.25	0.06 ± 0.56	0.03 ± 0.37	N/A	N/A
	First oviposition (cw)	32	36	36	N/A	N/A

**Table 3:** Influences of the grape variety on *Drosophila suzukii* behavioral preferences in dual-choice assays. X^2^- and *p*-values for the fixed effect term obtained from the likelihood ratio tests on linear mixed-effects models (LMMs) (*p*-values < 0.05 are highlighted in bold) and estimates for the variance (Var) explained by random effects as well as unexplained variance (residuals) with the number of observations (Obs) for each of these groups.

Combination Tested		Regent vs. Müller-Thurgau		Acolon vs. Müller-Thurgau		Pinot Noir vs. Pinot Blanc		Regent vs. Pinot Noir	
Fixed effects	*Df*	*Χ* ^2^	*p*	*Χ* ^2^	*p*	*Χ* ^2^	*p*	*Χ* ^2^	*p*
Location preference									
Grape variety	1	66.06	**<0.001**	26.14	**<0.001**	13.76	**<0.001**	4.62	**0.032**
Random effects		Var	Obs	Var	Obs	Var	Obs	Var	Obs
ID		0	30	0	10	0	10	0	40
Year: date		0	3					0.09	4
Year		0	2					0	2
Residuals		1.71	60	0.95	20	1.98	20	3.22	80
Oviposition preference									
Grape variety	1	16.95	**<0.001**	3.08	0.079	1.08	0.298	18.63	**<0.001**
Random effects		Var	Obs	Var	Obs	Var	Obs	Var	Obs
ID		0	30	0	10	0	10	0	40
Year: date		0	3					0	4
Year		5.19	2					4.43	2
Residuals		47.56	60	1.5	20	0	20	25.20	80

**Table 4:** Mean (±SE) adult emergence rate *of Drosophila suzukii* from eggs laid in berries of different grape varieties and conditions in dual-choice assays performed in 2017 (see Table 1); *p*-values < 0.05 are highlighted in bold (Mann–Whitney U test).

Test	Grape Variety	Berry Condition	Emergence Rate (%)	W	*p*
1	Regent	intact	8.7 ± 5.0	231	0.305
	Müller-Thurgau	intact	7.9 ± 4.4		
4	Regent	intact	19.5 ± 7.3	288	**0.003**
	Pinot Noir	intact	1.7 ± 1.6		
6	Müller-Thurgau	intact	1.7 ± 1.7	9	**0.001**
		damaged	26.1 ± 5.5		
7	Pinot Blanc	intact	5.0 ± 5.0	6.5	**<0.001**
		damaged	35.6 ± 4.3		

**Table 5:** Influences of berry condition on *Drosophila suzukii* behavioral (location and oviposition) preferences in dual-choice assays. X^2^- and *p*-values for the fixed effect term obtained from likelihood ratio tests on linear mixed-effects models (LMMs) (*p*-values < 0.05 are highlighted in bold) and estimates for the variance (Var) explained by random effects as well as unexplained variance (residuals) with the number of observations (Obs) for each of these groups.

Combination Tested		Intact vs. Damaged Berries					
		Pinot Noir		Müller-Thurgau		Pinot Blanc	
Fixed effects	*Df*	*Χ* ^2^	*p*	*Χ* ^2^	*p*	*Χ* ^2^	*p*
Location preference							
Berry condition	1	36.67	**<0.001**	6.00	**0.014**	2.80	0.095
Random effects		Var	Obs	Var	Obs	Var	Obs
ID		0	10	0	10	0	10
Residuals		0.55	20	2.07	20	2.4	20
Oviposition preference							
Berry condition	1	24.59	**<0.001**	17.17	**<0.001**	34.02	**<0.001**
Random effects		Var	Obs	Var	Obs	Var	Obs
ID		0	10	12.55	10	3.03	10
Residuals		3.85	20	101.78	20	42.12	20

## Supplementary Materials

The following are available online at https://www.mdpi.com/2075-4450/10/12/432/s1, Table S1: Number of sample sites, sample number and weekly mean (±SD) *Drosophila suzukii* infestation severity (number of eggs per berry sample in %) of grape varieties investigated for infestation in viticultural districts all across Baden-Wuerttemberg, southwestern Germany from 2015 to 2018. Varieties that were used in dual-choice assays are highlighted in bold. “N/A” = data not available.
