# Supplementary material for: Effects of Variety and Grape Berry Condition of Vitis vinifera on Preference Behavior and Performance of Drosophila suzukii
Source: Insects. 2019 Nov 30;10(12):432. doi: 10.3390/insects10120432 (PMC6955987; doi:10.3390/insects10120432)
Supplement: Supplementary file 1 [file insects-10-00432-s001.pdf]

Supplementary Materials:

Table S1: Number of sample sites, sample number and weekly mean (± SD) *Drosophila suzukii* infestation severity (number of eggs per berry sample in %) of grape varieties investigated for infestation in viticultural districts all across Baden-Wuerttemberg, southwestern Germany from 2015 to 2018. Varieties that were used in dual-choice assays are highlighted in bold. "N/A" = data not available.

	**2015**			**2016**			**2017**			**2018**		
	Sample sites	Sample number	Severity (%)	Sample sites	Sample number	Severity (%)	Sample sites	Sample number	Severity (%)	Sample sites	Sample number	Severity (%)
**Red varieties**												
**Acolon**	**8**	**32**	**0**	**20**	**97**	**3.57 ± 6.96**	**17**	**53**	**2.60 ± 7.23**	**14**	**76**	**0.04 ± 0.25**
Blauer Zweigelt	N/A	N/A	N/A	1	2	1.00 ± 1.00	N/A	N/A	N/A	N/A	N/A	N/A
Cabernet Cantor	N/A	N/A	N/A	2	7	0	1	1	0	2	18	0
Cabernet Carol	6	25	2.56 ± 10.95	5	20	1.00 ± 2.72	1	2	1 ± 1	N/A	N/A	N/A
Cabernet Cortis	5	16	0	10	38	2.21 ± 8.73	2	2	2 ± 2	2	22	0
Cabernet Dorsa	5	28	0.21 ± 1.11	16	69	1.52 ± 3.22	4	13	7.54 ± 15.91	6	70	0
Cabernet Jura	N/A	N/A	N/A	1	7	0	N/A	N/A	N/A	N/A	N/A	N/A
Cabernet Mitos	N/A	N/A	N/A	3	3	0	1	2	0	N/A	N/A	N/A
Dornfelder	10	48	8.56 ± 29.95	12	46	4.57 ± 10.92	11	33	22.36 ± 51.59	9	82	0.93 ± 2.30
Dunkelfelder	12	61	0.10 ± 0.43	16	65	4.03 ± 8.09	14	54	3.41 ± 7.69	11	134	0
Frühburgunder	1	9	0.22 ± 0.63	1	4	1.00 ± 1.73	1	2	0	1	10	0
Lemberger	4	26	0.92 ± 2.16	4	22	1.73 ± 3.63	5	18	2.22 ± 3.58	5	34	2.82 ± 6.25
Merlot	2	10	0.40 ± 1.20	1	5	0.40 ± 0.80	1	5	0	N/A	N/A	N/A
Monarch	2	9	1.33 ± 3.13	4	17	2.00 ± 3.29	1	1	0	N/A	N/A	N/A
**Pinot Noir**	**34**	**130**	**0.44 ± 1.67**	**77**	**201**	**0.29 ± 1.61**	**42**	**108**	**1.23 ± 6.87**	**26**	**119**	**0.03 ± 0.37**
Pinotin	1	1	0	1	2	0	1	1	26 ± 0	1	14	0
Portugieser	4	22	0.27 ± 0.69	2	7	0.29 ± 0.70	2	3	20 ± 22.63	2	16	2.75 ± 5.19
Prior	N/A	N/A	N/A	3	4	11.50 ± 19.92	1	1	0	N/A	N/A	N/A
**Regent**	**24**	**129**	**1.58 ± 5.47**	**48**	**199**	**3.03 ± 13.03**	**25**	**104**	**2.13 ± 6.82**	**23**	**127**	**0.06 ± 0.56**
Roter Gutedel	10	32	13.6 ± 27.44	9	31	0.91 ± 3.21	5	12	1.96 ± 3.35	6	58	0.14 ± 0.73
Roter Müller-Thurgau	1	7	27.24 ± 22.12	1	6	0	1	3	1.33 ± 1.89	1	8	0
Roter Muskateller	3	9	32.15 ± 54.42	13	20	0.30 ± 0.95	2	3	0	2	22	0.55 ± 1.72
Saint Laurent	N/A	N/A	N/A	1	1	0	N/A	N/A	N/A	N/A	N/A	N/A
Schwarzriesling	5	33	1.09 ± 2.96	5	22	1.09 ± 2.39	5	13	4.00 ± 7.65	5	34	0.35 ± 1.41
Syrah	1	7	0.29 ± 0.70	1	5	0	1	3	0	N/A	N/A	N/A
Tauberschwarz	N/A	N/A	N/A	1	4	0.50 ± 0.87	1	3	40.00 ± 42.71	1	4	0
Trollinger	8	53	2.83 ± 8.85	9	36	19.17 ± 28.77	9	23	68.35 ± 119.34	8	70	10.00 ± 19.21
**White varieties**												
Auxerrois	1	3	0	1	1	0	N/A	N/A	N/A	N/A	N/A	N/A
Cabernet Sauvignon	1	1	0	N/A	N/A	N/A	N/A	N/A	N/A	N/A	N/A	N/A
Gelber Muskateller	2	3	0	1	1	0	2	5	0	N/A	N/A	N/A

Gewürztraminer	3	16	0.13 ± 0.48	7	24	0.25 ± 1.20	3	9	0	2	20	0
Grüner Silvaner	1	6	0	N/A	N/A	N/A	N/A	N/A	N/A	N/A	N/A	N/A
Kerner	1	7	0	1	4	0	N/A	N/A	N/A	N/A	N/A	N/A
**Müller-Thurgau**	**3**	**11**	**0.30 ± 0.96**	**N/A**	**N/A**	**N/A**	**5**	**6**	**0**	**N/A**	**N/A**	**N/A**
**Pinot Blanc**	**1**	**5**	**0**	**2**	**2**	**0**	**N/A**	**N/A**	**N/A**	**N/A**	**N/A**	**N/A**
Pinot Gris	5	12	0	12	28	0	5	11	0	3	16	0
Solaris	2	10	0	6	20	0	2	6	0	2	14	0
Weißer Gutedel	1	1	0	N/A	N/A	N/A	N/A	N/A	N/A	N/A	N/A	N/A
Weißer Riesling	2	11	0	N/A	N/A	N/A	N/A	N/A	N/A	N/A	N/A	N/A
